# Temporal prediction error triggers amygdala-dependent memory updating in appetitive operant conditioning in rats

**DOI:** 10.3389/fnbeh.2022.1060587

**Published:** 2023-01-10

**Authors:** Tatiane Ferreira Tavares, José Lino Oliveira Bueno, Valérie Doyère

**Affiliations:** ^1^Laboratory of Associative Processes, Temporal Control and Memory, Department of Psychology, University of São Paulo, Ribeirão Preto, Brazil; ^2^Institut des Neurosciences Paris-Saclay – NeuroPSI CNRS, Université Paris-Saclay, Saclay, France

**Keywords:** negative prediction error, omission effect, reconsolidation, timing, basolateral amygdala

## Abstract

Reinforcement learning theories postulate that prediction error, i.e., a discrepancy between the actual and expected outcomes, drives reconsolidation and new learning, inducing an updating of the initial memory. Pavlovian studies have shown that prediction error detection is a fundamental mechanism in triggering amygdala-dependent memory updating, where the temporal relationship between stimuli plays a critical role. However, in contrast to the well-established findings in aversive situations (e.g., fear conditioning), only few studies exist on prediction error in appetitive operant conditioning, and even less with regard to the role of temporal parameters. To explore if temporal prediction error in an appetitive operant paradigm could generate an updating and consequent reconsolidation and/or new learning of temporal association, we ran four experiments in adult male rats. Experiment 1 verified whether an unexpected delay in the time of reward’s availability (i.e., a negative temporal prediction error) in a single session produces an updating in long-term memory of temporal expectancy in an appetitive operant conditioning. Experiment 2 showed that negative prediction errors, either due to the temporal change or through reward omission, increased in the basolateral amygdala nucleus (BLA) the activation of a protein that is critical for memory formation. Experiment 3 revealed that the presence of a protein synthesis inhibitor (anisomycin) in the BLA during the session when the reward was delayed (Error session) affected the temporal updating. Finally, Experiment 4 showed that anisomycin, when infused immediately after the Error session, interfered with the long-term memory of the temporal updating. Together, our study demonstrated an involvement of BLA after a change in temporal and reward contingencies, and in the resulting updating in long-term memory in appetitive operant conditioning.

## 1. Introduction

Memories are dynamic instead of static, and once consolidated, they can be updated through additional experience. Thus, the brain is continually encoding, consolidating, and reconsolidating information from the environment (Dudai, 2012). After the initial encoding of new information, a consolidated memory is subject to modification through subsequent reminders and interference. Thus, each time a memory is retrieved, it goes through changes because it is experienced in a different moment and with different characteristics. This memory updating process includes both a different experience that can be connected to the initial memory (old memory), as well as a second learning experience (new memory) (Alberini, 2011).

The processes by which memories are updated have been linked to the cellular mechanisms of memory reconsolidation of old memories and consolidation of the new ones. In certain conditions, the old memory can be modified (enhanced or disrupted) (Doyère et al., 2007; Choi et al., 2010; Lee, 2010; Debiec et al., 2011; Tian et al., 2011), suggesting that the destabilization and subsequent reconsolidation may be a process that enables the memory updating by connecting new information into previous memories (Lee, 2009; Díaz-Mataix et al., 2013, 2014; Flavell and Lee, 2013). In contrast, a repeated series of reminding events after initial encoding constitutes a training inducing new learning, with new information consolidating at a cellular level and connecting among already old memories (Fernández et al., 2016). Thus, memories can be reactivated through the presentation of cues (reminders) already present at the time of acquisition, and the interaction between the reactivation and the characteristics of the initial memory determines whether or not memory retrieval could induce memory reconsolidation and/or new learning.

Memories undergo a series of changes, transforming the unstable state into one that is stable and long lasting. During this transforming state, the memory is sensitive to several differing types of interference, including behavioral or cognitive interference, and pharmacological or molecular manipulations (Exton-McGuinness et al., 2015). The neurobiological mechanisms are very similar during reconsolidation and consolidation processes, although not identical (Lee et al., 2004; Alberini, 2005; Alberini and LeDoux, 2013; Li et al., 2013). In order to persist in the longer term, newly acquired memories consolidate into stable traces by mechanisms requiring protein synthesis and synaptic plasticity (McGaugh, 2000; Fernández et al., 2016). Molecular interference, such as with protein synthesis inhibitors, can affect both consolidation and reconsolidation processes (Boccia et al., 2004; Wang et al., 2005; Duvarci et al., 2008). One factor thought to be critical in defining memory reconsolidation is whether amnesic agents applied after memory reactivation impair the original memory trace, rather than reactivation to create a new memory that undergoes consolidation. If reactivation generates a new trace, then blocking this trace should not interfere with the old memory (Debiec et al., 2002; Alberini, 2011). Insights into how the brain determines whether a memory trace should be reconsolidated or an additional memory trace be formed are essential for our understanding of the basis of learning and memory.

Behavioral interference can be achieved by violating an expected association inducing a surprise and mismatches, and producing an error in prediction (Rescorla and Wagner, 1972; Pearce and Mackintosh, 2010; Dudai, 2012; Exton-McGuinness et al., 2015). Some evidences indicate that the prediction error, a discrepancy between the actual and expected outcomes (Rescorla and Wagner, 1972; Pearce and Hall, 1980), drives the updating of memories, whether through reconsolidation or consolidation (Pedreira et al., 2004; Sevenster et al., 2012, 2013, 2014; Exton-McGuinness et al., 2015). In this context, an unexpected outcome or surprising event induces a prediction error (mismatch–reactivation) which triggers the reconsolidation process, inducing memory updating and adjustment for future predictions. However, repeated events of mismatching constitute a training, inducing new learning (Fernández et al., 2016).

Pavlovian conditioning is the reference paradigm for the study of associative learning between two stimuli, the conditioned stimulus (CS) and the unconditioned stimulus (US), and it has been employed in most studies of prediction error to explore the processes of reconsolidation and/or new learning (Lewis, 1979; Nader et al., 2000; Nader, 2003; Finnie and Nader, 2012). For example, within the context of the Pearce and Hall (1980) model of associative learning, studies have shown increased learning after CS omission (Holland and Gallagher, 1993; Lee H. J. et al., 2006; Lee H. J. et al., 2010). If, after intensive training to a light-tone serial compound, the tone is omitted on some trials, rats later learn light-food associations faster than if only light-tone trials were presented. Thus, violating the light-tone expectancy by presenting the light alone retrieves that associability, allowing the light to acquire association with the food faster later (Holland and Gallagher, 1993; Holland and Schiffino, 2016), thus driving a new learning through the omission of the CS.

In the reconsolidation framework, the most frequently used reminder session is with the omission of the US after the cue presentation, or CS, previously associated. The process is typically revealed by showing an impairment of the initial memory during the test, when using pharmacological or behavioral tools after the reminder (Lee, 2009). Studies have primarily used aversive Pavlovian conditioning paradigms, including contextual or auditory fear conditioning, and conditioned taste aversion (Nader et al., 2000; Debiec et al., 2002; Kida et al., 2002; Eisenberg et al., 2003). Interestingly, recent studies have explored the relevance of interval timing in triggering prediction error and updates in studies of Pavlovian aversive conditioning (Díaz-Mataix et al., 2013; Dallérac et al., 2017). Díaz-Mataix et al. (2013) showed that a change in the temporal relationship between CS and US during reactivation triggers synaptic plasticity and reconsolidation of an aversive memory in the lateral nuclei of the amygdala. Memory reconsolidation only occurred when a temporal mismatch between the expected and the actual time of arrival of the US was detected. It is clear that temporal variables have an effect on learning, and it seems that prediction errors can lead to alterations in temporal expectancies. Some studies indicate the importance of timing in the reward prediction (Roberts, 1981; Grace and Nevin, 2000; Doughty and Richards, 2002; Ludvig et al., 2007; Galtress and Kirkpatrick, 2009). This important role of time in associative learning has been incorporated in temporal difference learning models (Sutton and Barto, 1981), and is the basis of the prediction error detection studies in which the omission of the CS or US is detected at the neural level at the time it is expected to arrive (Schultz, 1998; Díaz-Mataix et al., 2014).

While prediction error is a critical factor in memory updating, reconsolidation and/or new learning, in Pavlovian conditioning (Debiec and Ledoux, 2004; Duvarci and Nader, 2004; Alberini, 2005; Lee H. J. et al., 2006; Lee J. L. et al., 2006; Lee, 2008), it is still questioned whether memory updating when acquired in operant conditioning could be driven by this process (Vousden and Milton, 2017; Piva et al., 2020). Reconsolidation of operant conditioning, when an action of the subject is associated with appetitive reinforcements, like natural rewarding stimuli (water, sucrose) or drugs of abuse (cocaine and nicotine), has recently been investigated. Although some studies demonstrated reconsolidation (Exton-McGuinness et al., 2014, 2019; Tedesco et al., 2014; Exton-McGuinness and Lee, 2015; Sorg et al., 2015; Exton-McGuinness and Milton, 2018), others failed to show destabilization and subsequent reconsolidation of operant appetitive memory (Hernandez et al., 2002; Hernandez and Kelley, 2004; Mierzejewski et al., 2009; Exton-McGuinness et al., 2014). The successful studies showed a decreased operant performance at test in the reactivation group after an amnestic manipulation. In the study of Tedesco et al. (2014), for example, they demonstrated destabilization of an operant conditioning using a non-reinforced reactivation session, and impairing of reconsolidation with the N-methyl-D-aspartate receptor (NMDAR) antagonist (±)-5-methyl-10,11-dihydro-SH-dibenzo[a,d]cyclohepten-5,10-imine maleate (MK-801). In addition, Exton-McGuinness et al. (2014) investigated whether a change of reward contingency, rather than non-reinforcement, would destabilize a well-learned operant conditioning. In their study, rats were trained for 10 days to lever press for food reinforcement on a fixed-ratio-1 (FR1) schedule, where each lever press provides one reward delivery. Administration of MK-801 impaired reconsolidation only when administered before the change in contingency to a variable-ratio-20 schedule. Disruption of reconsolidation caused a reduction in lever pressing performance, further to a loss of sensitivity to contingency change, suggesting that it was the instrumental component of behavior that was disrupted.

Importantly, the differential results in the reconsolidation in operant conditioning studies are highlighting the experimental boundary conditions under which reconsolidation has to take place, with precise parameters of the reactivation session as a key factor in determining whether reconsolidation or consolidation processes occur (Exton-McGuinness et al., 2015; Piva et al., 2020).

Thus, the question of the neural underpinnings for memory updating in the operant conditioning and of the controlling parameters is an open question. The literature suggests that the amygdala may be involved in the processing of prediction error in Pavlovian conditioning (Lee H. J. et al., 2006; Lee, 2008), in the processing and storage of the CS–US time interval (Díaz-Mataix et al., 2013, 2014; Dallérac et al., 2017), and in the unexpected reward omission in operant conditioning (Henke, 1973; Henke and Maxwell, 1973; Bueno et al., 2012; Judice-Daher et al., 2012; Tavares et al., 2014). In addition, studies demonstrated that basolateral amygdala (BLA) neurons increase their firing rates to prediction error (Bermudez and Schultz, 2010; Roesch et al., 2010, 2012; Schultz, 2015). Moreover, anisomycin infusion, an amnesic agent, into the BLA disrupts memory consolidation in several tasks, such as trace and delay fear conditioning, odor aversion memory and devaluation of reward (Wang et al., 2005; Desgranges et al., 2008; Kwapis et al., 2011). However, while the temporal amygdala-dependent memory updating has been demonstrated in Pavlovian aversive conditioning (Díaz-Mataix et al., 2013; Dallérac et al., 2017), it is not known whether temporal parameters play the same role in prediction error and memory updating in appetitive operant conditioning in an amygdala-dependent manner. An unexpected change in temporal parameters could interfere not only with the associative strength of the old memory, but may also trigger an updating of the temporal expectancy that was built in the old memory. Disrupting the temporal association via amnesic agents could affect the original temporal memory trace (old memory) that undergoes reconsolidation, or the new temporal memory trace (new memory) that undergoes consolidation. Thus, temporal error detection could generate, with reconsolidation and/or consolidation processes, a long-lasting new temporal expectancy.

To explore whether a temporal prediction error in an appetitive operant conditioning could generate an updating in expectancy through reconsolidation and/or new learning processes in an amygdala-dependent manner, we ran four experiments in rats. Experiment 1 verified whether an unexpected delay in the time of reward’s availability in a single session produces an updating in long-term memory of temporal expectancy in an appetitive operant conditioning. Experiment 2 verified whether prediction error, either due to the temporal change or through reward omission, increased in the BLA the activation of a protein that is critical for memory formation. Experiment 3 and 4 evaluated if a protein synthesis inhibitor (anisomycin), when infused before or after the Error session, interfered with the temporal expectancy through reconsolidation and/or consolidation processes.

## 2. Materials and methods

### 2.1. Experiment 1

#### 2.1.1. Subjects

Adult male Sprague-Dawley rats provided by Harlan (France) and weighing 250–300 g at the beginning of the experiments were used. Throughout the experiments, the animals were housed in pairs, in Plexiglas cages, in the laboratory colony room on a 12 h/12 h light/dark schedule (lights on 8:00 AM to 8:00 PM). The rats were maintained on a food restriction schedule at 85% of their *ad libitum* body weight. The rats were deprived for 23 h before the beginning of each session. Water was freely available in their cages. All procedures were in accordance with the guidelines of the EU, CNRS, and the French Agricultural and Forestry Ministry (2010/63/UE, French decree article R-214-89) and were submitted for approval to the Ethics Committee N°59.

#### 2.1.2. Apparatus

Behavioral training took place in a set of four identical conditioning chambers (30 cm × 25 cm × 30 cm, Coulbourn Instruments, USA). Each chamber was equipped with a grid floor, a lever, a food magazine connected to a pellet dispenser and a speaker, all placed in a sound attenuating enclosure with a ventilation fan (60 dB background noise). Behavioral protocols were controlled by Graphic State software (Coulbourn Instruments, USA).

#### 2.1.3. Pre-training

Pre-training was carried out over two sessions. In the first session, each rat was trained to receive 30 single pellet foods (45 mg, BioServ, USA) at variable intervals (40, 60, and 80 s). The following session consisted of continuous reinforcement (CRF training, with a single pellet delivered after each lever press), for a total of 100 lever presses. Each session lasted a maximum of 1 h. After pre-training, the animals were submitted to three phases: training, error session and reinforcement omission effect (ROE) test. The same Pre-training was carried out for the Experiments 2, 3, and 4.

#### 2.1.4. Training

Twenty-seven rats were trained to respond on a Fixed-Interval (FI) 6 s with Limited Hold (LH) 12 s schedule signaled by a 18-s tone (FI 6 s LH 12 s). In this schedule, a tone is played for 18 s and, from 6 s after the tone onset, a reward is available during the last 12 s, but only the first lever press during this last 12 s period results in delivery of one pellet (Bueno et al., 2012; Judice-Daher et al., 2012; Tavares et al., 2014, 2019). Fifteen sessions were performed. Rats received 20 trials per session with variable intertrial intervals (30, 60, 90, and 120 s) and all correct responses were reinforced (100% Reinforcement). At the end of each session, the rats were returned to their cages and given sufficient food to maintain their planned body weight schedule. The same Training was carried out for the Experiments 2, 3, and 4.

#### 2.1.5. Error session

For a single Error session, rats were divided in two groups: No Change group (*n* = 14) had the same Training schedule, while Temporal Change group (*n* = 13) had a change in the temporal schedule by delaying the time of reward’s availability, where the FI time was changed from 6 to 12 s to obtain the reward (prediction error). So, in this new association, after 12 s of tone, the reward was available during the last 6 s, but only the first lever press in this last period was reinforced (FI 12 s LH 6 s signaled schedule of reinforcement). The same Error session was carried out for the Experiments 3 and 4.

#### 2.1.6. Reinforcement omission effect (ROE) test

Twenty-four hours later, both groups were submitted to the same schedule as in Training, but the reward was omitted after the correct response in 50% of the trials, so 10 trials were reinforced and 10 trials were non-reinforced. The reinforced or non-reinforced trials were pseudorandomly distributed during the session, using the criterion of up to two identical subsequent trials. All animals got exactly the same session. The same ROE test was carried out for the Experiments 3 and 4.

### 2.2. Experiment 2

The following procedures were the same as in Experiment 1: Subjects, apparatus, pre-training and training. After training, animals were submitted to one error session followed by perfusion for immunohistochemistry analyses.

#### 2.2.1. Training

Training was the same as of Experiment 1, except that 29 rats were used.

#### 2.2.2. Error session

For a single Error session, rats were divided in three groups: No Change group (*n* = 9) and Temporal Change group (*n* = 10) were submitted to the same schedule as in the Experiment 1; ROE group (*n* = 10) was submitted to the same schedule as during Training but the reward was omitted after the correct response in half of the trials (50% R and 50% N). Ninety minutes from the beginning of the Error session, the animals were sacrificed and their brain taken for immunohistochemistry analyses.

#### 2.2.3. Immunohistochemistry

For activity-regulated cytoskeletal (Arc) immunostaining, rats were perfused at 90 min from beginning of the Error session. After a rapid deep anesthesia with an overdose of pentobarbital (i.p., Dolethal, 1 mL/100 g; diluted 1/3 in 0.9% NaCl), rats were transcardially perfused with 300 ml phosphate-buffered saline (PBS) followed by 300 ml of ice-cold 4% paraformaldehyde (PFA) in 0.1 M phosphate buffer. Brains were removed, post-fixed overnight in 4% PFA, and placed in a cryoprotecting solution composed of 30% glycerol and 0.1% sodium azide in 0.1 M phosphate buffer. Free-floating sections (40 μm) containing the regions of interest were cut using a freezing microtome. Every sixth sections from antero-posterior amygdala (Paxinos and Watson, 2007) were processed for Arc immunoreactivity. After blocking in PBS containing 1% bovine serum albumin-0.1% Triton X-100, slices were incubated overnight at room temperature in anti-Arc antibody (mouse monoclonal sc-17839, 1:500; Santa Cruz Biotechnology) in PBS containing 1% BSA-0.1% Triton X-100. After extensive washes in PBS, tissue sections were incubated with secondary antibody (Vectastain Anti-mouse IgG, biotinylated antibody 1:500; Elite PK-6102) in PBS-1% BSA. This was finally followed by washes and processing using the VectaStain Elite ABC kit (Vector Laboratories) and development in DAB peroxidase substrate for 5 min. In addition, some sections were also processed for negative tests in which the primary antibody was not included in the protocol, in order to verify the specificity of the Arc antibody. Sections were mounted on electrostatic slides and coverslipped with DPX mounting medium. Images at ×4 were collected using an Olympus BX60 microscope (Leica Microsystems, Germany) equipped with a CoolSNAP camera (Roper Scientific, USA) and Openlab software (Improvision, UK). Cell counting was performed in a defined region of interest (240 × 220 pixels) using Image J.

### 2.3. Experiment 3

The following procedures were the same as in Experiment 1: subjects, apparatus, pre-training, training, error session and ROE test. However, after Training, the animals were submitted to Surgery and after their recovery, they had a retraining phase before the Error session.

#### 2.3.1. Training

Training was the same as in Experiment 1, except that 43 rats were used. After Training, rats were submitted to surgical implantation of cannulae.

#### 2.3.2. Surgery

Stereotaxic surgery was conducted under ketamine (75 mg/kg, i.p.) and domitor (50 mg/kg, i.p.) anesthesia. Rats were implanted bilaterally with 26-gauge stainless guide cannulae (PlasticsOne, Roanoke, VA, USA) aimed at the basolateral complex of the amygdala (BLA). All coordinates were taken from Paxinos and Watson (2007). Coordinates for intra-BLA were: 3.0 mm posterior to bregma, 5.2 mm lateral to the midline and 7.6 mm ventral to the skull surface. The guide cannulae were fixed to screws in the skull using acrylic dental cement. A dummy cannula was inserted into each guide cannula to prevent clogging. Postsurgical analgesic (Tolfedine (0.01 ml/100 g, i.p.) was given after all surgeries. Rats had at least 1 week to recover before the return to behavioral procedures.

#### 2.3.4. Retraining–100% R

After 1 week of recovery, rats were submitted to Retraining (7 sessions), following the same procedure as in Training. After Retraining, an Error session was performed.

#### 2.3.5. Error session

Error session was the same in Experiment 1 for the Temporal Change group, but rats were divided in two groups: Vehicle (*n* = 21) and Anisomycin (*n* = 22). The animals received vehicle or anisomycin infusion 10 min before the Error session, followed 24 h later by the ROE Test session.

#### 2.3.6. Drug infusions

Using an infusion pump, anisomycin or an equivalent volume of artificial cerebrospinal fluid (ACSF) was injected bilaterally into the BLA at a rate of 0.25 μl/min. Following drug infusion, injector cannulae were left in place for an additional minute to allow diffusion of the drug away from the cannula tip. Anisomycin (Sigma, St. Louis, MO, USA) was dissolved in equimolar HCL, diluted with ACSF, and adjusted to pH 7.4 with NaOH. The drug concentration was 125 μg/μl. The volume of anisomycin (or ACSF) infused intra-BLA was 0.5 μl for each side.

#### 2.3.7. Histology

At the termination of the experiment, rats were euthanized by an overdose of sodium pentobarbital (150 mg/kg, i.p.). Their brains were removed, sectioned at 40 μm thickness and examined with light microscopy for cannula penetration. After histological verification, only animals that had both cannulae into the BLA (including anterior and posterior areas), ranging from −2.52 to −3.24 (Paxinos and Watson, 2007) were included in the data analysis. After histological analysis, five rats of the Vehicle group and seven rats of the Anisomycin group were discarded because of incorrect placements of the cannula tip. The remaining rats of Vehicle (*n* = 16) and Anisomycin group (*n* = 15) were included in the behavioral analysis.

### 2.4. Experiment 4

All procedures were the same as in Experiment 3 (subjects, apparatus, pre-training, training, surgery, retraining, error session and test), except: 22 rats were used; Vehicle (*n* = 11) and Anisomycin (*n* = 11) groups received infusion immediately after the error session. After histological analysis, three rats of the Vehicle group and four rats of Anisomycin group were discarded because of incorrect placements of the cannula tip. The remaining rats of Vehicle (*n* = 8) and Anisomycin group (*n* = 7) were included in the behavioral analysis.

## 3. Behavioral and histological analyses

### 3.1. Behavioral analyses

Response rates were analyzed during the 18 s tone in different phases: last 2 sessions of Training (Experiments 1, 2, 3, and 4); last 2 sessions of Retraining (Experiments 3 and 4), the Error session (Experiments 1, 2, 3, and 4) and the ROE test session (Experiments 1, 3 and 4). The average of lever presses from each rat was calculated dividing the total number of lever presses for each second performed in one session, by the number of trials (20 trials in 100% reinforcement condition, or 10 trials R and 10 trials *N* in 50% reinforcement condition). The data of the subjects were grouped to obtain the lever presses average for each 2 s during the 18 s tone (2, 4, 6, 8, 10, 12, 14, 16, and 18 s). Data were analyzed by two-way analyses of variance (ANOVA) with repeated measures using Jasp (0.16.0.0) statistical software. Significant effects in the ANOVA (*p* < 0.05) were followed by the *post-hoc* test (Bonferroni).

### 3.2. Histological analyses

In Experiment 2, the number of Arc-positive cells in basolateral (BLA), lateral (LA) and central (CeA) amygdala nuclei was quantified by calculating the density of particles analyzed using NIH ImageJ software. One coronal section for the posterior part of the amygdala ranging from −2.52 to −3.24 for each rat was analyzed. After threshold adjustments, the mean of bilateral values of density to each area were calculated, so the mean of each group was grouped. Data were analyzed by two-way analyses of variance (ANOVA) using Jasp statistical software (0.16.0.0). Significant effects in the ANOVA (*p* < 0.05) were followed by the *post-hoc* test (Bonferroni).

## 4. Results

### 4.1. Temporal prediction error generates an updating in long-term memory

**FIGURE 1:**
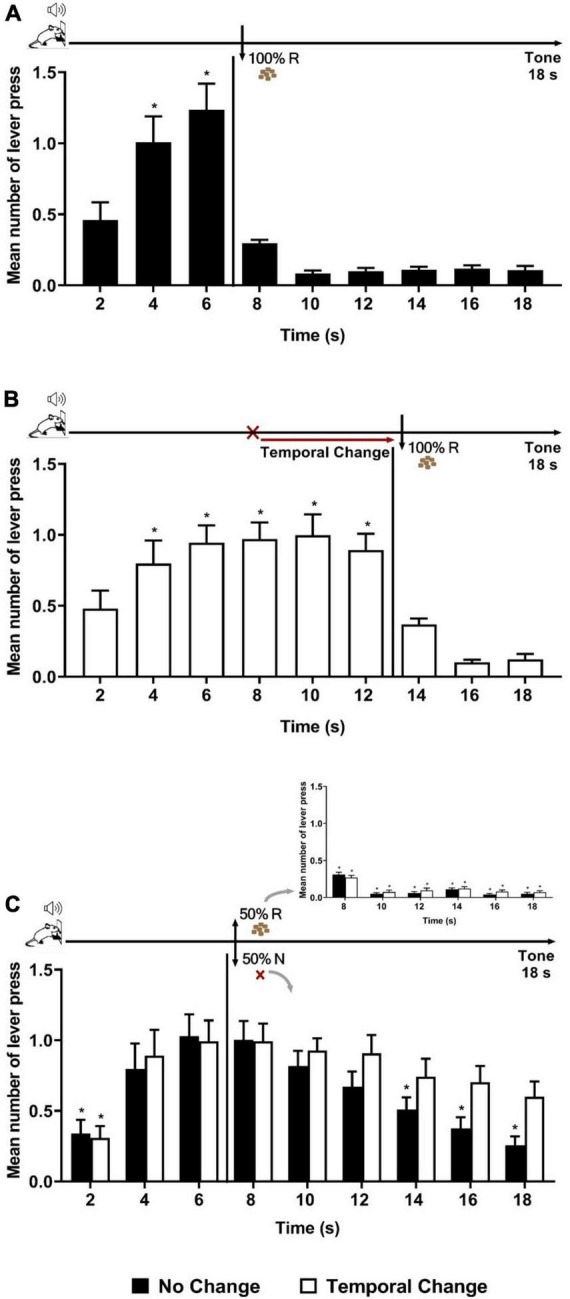
Temporal prediction error generated a long-lasting memory updating of temporal expectancy. Responses average (+ SEM) during the 18-s tone, per 2 s, for No Change (Black columns; *n* = 14) and Temporal Change (White Columns, *n* = 13) groups. **(A)** Error session of No Change Group during FI 6 s LH 12 s signaled schedule (*p < 0.05, *post hoc* intra-group differences from 2 s). **(B)** Error session of Temporal Change group during FI 12 s LH 6 s signaled schedule during which the FI time was changed from 6 to 12 s to obtain the reinforcement (*p < 0.05, *post hoc* intra-group differences from 2 s). **(C)** Test session of reinforcement omission effect (ROE) during FI 6 s LH 12 s signaled schedule in which the reinforcement was omitted in 50% of trials (*p < 0.05, *post hoc* intra-group differences from 6 s). FI: fixed-interval; LH: limited hold; R: Reinforcement; N: non-reinforcement.

We first aimed to verify whether a single session (20 trials) with an unexpected delay in the time of reward’s availability (Error session) produces an updating of temporal expectancy in long-term memory 24 h after in an appetitive operant conditioning (temporal expectancy of reward). We compared two groups of rats: No Change and Temporal Change. The animals were trained to a 18-s tone during which, after 6 s from tone onset, a reward was available for the last 12 s, but only the first lever press during this last period was reinforced, in a 100% Reinforcement condition [fixed interval (FI) 6 s limited-hold (LH) 12 s schedule of reinforcement]. The two groups were then formed, with similar performance during Training: ANOVA of lever presses per 2-s time bins confirmed no significant effect of groups [*F* (1, 22) = 0.251, *p* = 0.620], nor group × time interaction [*F* (8, 200) = 0.191, *p* = 0.992], but a significant effect of time [*F* (8, 200) = 72.115, *p* < 0.001]. *Post hoc* analyses indicated that the number of lever presses increased at 4 and at 6 s compared to time 2 s, showing good temporal control by the reward. After Training, the animals underwent one so-called “Error session,” also in a 100% Reinforcement condition. Animals of the No Change group experienced the same schedule (FI 6 s) as in Training (Figure 1A, top diagram), whereas the others (Temporal Change group) experienced a change in the temporal association, where the FI time was delayed from 6 to 12 s to obtain the reinforcement (Figure 1B, top diagram). In this new temporal association, it is only after 12 s of tone that the reinforcement was available, and the first lever press in the last 6 s period was reinforced (FI 12 s LH 6 s signaled schedule of reinforcement). As expected, animals in the No Change group presented the same performance as in Training (Figure 1A). In contrast, animals in the Temporal Change group updated their responses to the new temporal association to obtain the reinforcement (Figure 1B). *Post hoc* analyses indicated that the number of lever presses from 4 to 12 s was increased compared to time 2 s, showing an updated temporal control as a function of new time of reward’s arrival.

Twenty-four hours after the Error session, both groups were submitted to a ROE test, during which the same schedule of reinforcement as in Training (FI 6 s) was applied, but with only 50% of the trials reinforced (Figure 1C inset) in order to observe during the non-reinforced trials the reward expectancy beyond the FI time. The ANOVA analysis of the full 18 s tone during the non-reinforced trials revealed a significant group × time interaction [*F* (8, 176) = 2.206, *p* = 0.029] and a time effect [*F* (8, 176) = 16.862, *p* < 0.001], with no significant effect of group [*F* (1, 22) = 0.161, *p* = 0.692]. The significant interaction indicates differential temporal pattern between groups. *Post hoc* analyses showed that the number of lever presses increased at 4 s and at 6 s compared to time 2 s in both groups. Both groups expressed the ROE [with responses rates higher after non-reinforcement (N) than after reinforcement (R)]. During the N trials, as expected, the animals of the No Change group showed a maximum expectancy around the FI (6 s) time with a reduction of lever pressing reaching significance from 14 s onward (14, 16, and 18 s) (Figure 1C). In contrast, the animals from the Temporal Change group kept their lever presses at a high level for the remaining duration of the tone [no significant difference between the time 6 s and any of the following times (8, 10, 12, 14, 16, and 18 s)]. These results indicate that the change from 6 to 12 s in the FI schedule during the Error session produced an updating of reward temporal expectancy in long-term memory that was evident 24 h later during the N trials. Thus, a single session of temporal prediction errors, with a new temporal association (12 s), generated a long-lasting memory updating of temporal expectancy.

### 4.2. Basolateral amygdala nuclei activation by prediction error

During the Error session, either a change in the time of reward’s availability (from 6 to 12 s) and/or the omission of reward’s availability at 6 s can be detected and may trigger plasticity. At the molecular level, Arc genes are critical for long-term synaptic plasticity, and for adaptive functions such as long-term memory formation. Thus, as long-term memory formation requires new gene transcription and protein production to stabilize recent changes, Experiment 2 assessed whether the basolateral amygdala nuclei (BLA) would detect the temporal change and/or reinforcement parameters by analyzing Arc activation using immunohistochemistry.

**FIGURE 2:**
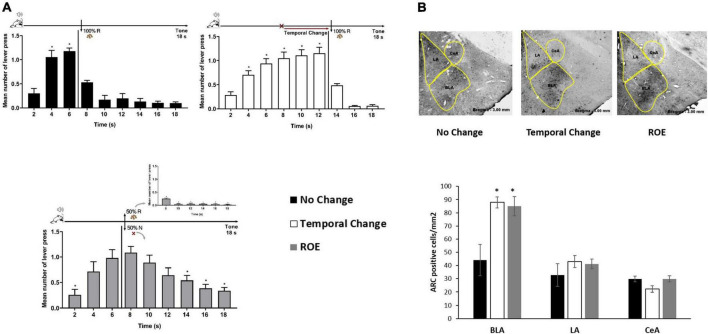
Basolateral amygdala nuclei are activated by the prediction error due to a change in the temporal rule and reinforcement omission. **(A)** Error session: responses average (+ SEM) during the 18 s tone, per 2 s, for three groups: No Change (Black columns; *n* = 9) during FI 6 s LH 12 s signaled schedule (*p < 0.05, *post hoc* intra-group differences from 2 s); Temporal Change (White columns; *n* = 10) during FI 12 s LH 6 s signaled schedule during which the FI time was delayed to 12 s to obtain the reward (*p < 0.05, *post hoc* intra-group differences from 2 s); reinforcement omission effect (ROE) (Gray columns; *n* = 10) during FI 6 s LH 12 s signaled schedule during which the reward was omitted in 50% of trials (*p < 0.05, *post hoc* intra-group differences from 6 s). **(B)** Activity-regulated cytoskeletal (ARC) activation: Quantification of the number of Arc-positive cells per square millimeter (mean ± SEM) across basolateral (BLA), lateral (LA) and central (CeA) posterior amygdala nuclei of No Change (Black columns), Temporal Change (White columns) and ROE (Gray columns) groups in animals perfused 90 min from the beginning of the Error session. Arc expression is increased in the BLA when the time to reward was changed from 6 to 12 s, as well as when the reinforcement was omitted (*p < 0.05, *post hoc* differences from No Change group). FI, fixed-interval; LH, limited hold; R, Reinforcement; N, non-reinforcement.

Rats trained to FI 6 s LH 12 s signaled schedule of reinforcement were distributed into three groups (No Change, Temporal Change and ROE groups). The behavior analysis confirmed that the animals presented similar performance during training as in the Experiment 1, with a significant effect of time [*F* (8, 208) = 145.219, *p* < 0.001], but no significant effect of group [*F* (2, 26) = 0.073, *p* = 0.930], nor group × time interaction [*F* (16, 208) = 0.183, *p* = 1.000]. Then, the animals went through an Error session, as in Experiment 1 for two groups (No Change and Temporal Change), while a third group underwent the session under a partial ROE schedule with no change in the FI time. *Post hoc* analyses showed that, as expected, the animals in the No Change group presented similar performance as in Training, the animals in the Temporal Change group updated their responses to the new temporal FI schedule (12 s) for reinforcement, and the animals in the ROE group expressed the ROE with a peak of responding in non-reinforced trials around 6 s (Figure 2A).

Ninety minutes from the beginning of the Error session, the animals were sacrificed under PFA perfusion and their brain taken for immunohistochemistry analyses of Arc-positive cells in the BLA, LA, and CeA amygdala nuclei. The ANOVA revealed a group effect in the number of Arc-positive cells [*F* (2, 23) = 6.789, *p* < 0.05], with a higher level of activation in Temporal Change and ROE groups compared to the No Change group in the BLA. However, in LA or CeA, no group effect was observed [*F* (2, 23) = 0.509, *p* = 0.608] and [*F* (2, 23) = 0.363, *p* = 0.700], respectively, with no difference in the level of activation in Temporal Change and ROE groups compared to the No Change group (Figure 2B). These data indicate that a protein that is critical for memory formation (Arc) was activated after detection of a prediction error, either due to the temporal change or through the reward omission during the ROE session, and only in the BLA.

### 4.3. Anisomycin infusion into the BLA affects memory updating and responding when a prediction error is detected

The next question Experiment 3 addressed was whether plasticity in the BLA is necessary for the updating of temporal expectancy, when a temporal prediction error is detected. We thus tested the impact of a protein synthesis inhibitor (anisomycin) when infused into the BLA before the Error session. The same Training, Error session and ROE phases as of the Temporal Change group in Experiment 1 were applied, except that the animals underwent a cannula implantation surgery after Training, followed by Retraining after recovery (7 sessions). Two groups of animals were then formed for them to receive a single infusion of anisomycin or vehicle into the BLA 10 min before the Error session.

**FIGURE 3:**
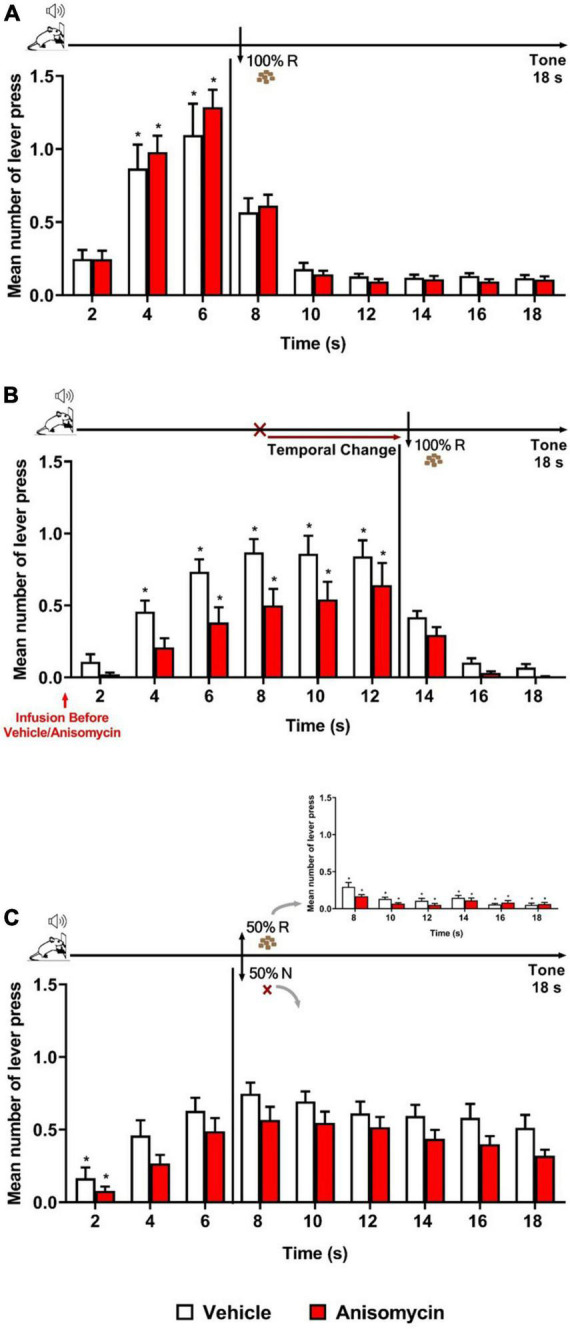
Anisomycin infusion before the Error session affected responding when a temporal prediction error was detected. Responses average (+SEM) during the 18 s tone, per 2 s, for Vehicle (White columns; *n* = 15) and Anisomycin (Red columns; *n* = 16) groups. **(A)** Retraining session during FI 6 s LH 12 s signaled schedule (*p < 0.05, intra-group differences from 2 s). **(B)** Error session of Temporal Change during FI 12 s LH 6 s signaled schedule during which the time was changed from 6 to 12 s to obtain the reward, and the vehicle or anisomycin were infused in the BLA 10 min before the session (*p < 0.05, intra-group differences from 2 s). **(C)** Reinforcement omission effect (ROE) Test session under FI 6 s LH 12 s signaled schedule during which the reward was omitted in 50% of trials (*p < 0.05, intra-group differences from 6 s). FI, fixed-interval; LH, limited hold; R, Reinforcement; N, non-reinforcement.

The results showed that both groups (Vehicle and Anisomycin) presented similar performance during Training and Retraining as in the previous experiments. During Retraining, the ANOVA revealed no significant effect of group [*F* (1, 29) = 0.017, *p* = 0.896], nor group × time interaction [*F* (8, 232) = 0.675, *p* = 0.713], but a significant effect of time [*F* (8, 232) = 49.205, *p* < 0.001] (Figure 3A). However, during the Error session under the FI 12 s LH 6 s schedule (when the time to obtain the reward was changed from 6 to 12 s), both groups updated their responding to the new reward time availability, but differentially, and animals in the Anisomycin group presented lower response rates than those in the Vehicle group (Figure 3B). The ANOVA confirmed a significant effect of group [*F* (1, 29) = 5.976, *p* < 0.05], time [*F* (8, 232) = 43.326, *p* < 0.01] and group × time interaction [*F* (8, 232) = 1.956, *p* < 0.05]. However, *post hoc* analyses never indicated any difference between groups at any time point, thus it was considered globally. The differential temporal pattern of responding to each group was evidenced with *post hoc* analyses indicating that the number of lever presses increased from 4 s and onward when compared to time 2 s for the animals of the Vehicle group, but only from 6 s and onward for those of the Anisomycin group.

Twenty-four hours after the Error session, the animals underwent the ROE test while back to the FI 6 s LH 12 s schedule. The analysis of the number of lever presses during the non-reinforced trials showed no significant effect of group [*F* (1, 29) = 3.315, *p* = 0.079] or group × time interaction [*F* (8, 232) = 0.290, *p* = 0.969], but a significant effect of time [*F* (8, 232) = 19.728, *p* < 0.01]. *Post hoc* analyses indicated that both groups exhibited the ROE to the same level, and that the temporal expectancy response dynamic was similar for both groups, with no significant decay in responding from 6 s until the end of the 18 s tone during non-reinforced trials (Figure 3C). Thus, although there was a trend for lower responding in the Anisomycin group, the differential temporal updating observed during the Error session was no longer visible at long-term. This may suggest that anisomycin in the BLA disrupted neither reconsolidation of the old temporal memory, nor the consolidation of the new updated temporal memory. Alternatively, as we do not know the time window of consolidation/reconsolidation sensitivity in our task and the anisomycin was infused before the 30-min session, its effects may have faded by the time reconsolidation or consolidation mechanisms are still active in the hours after the session. However, it also remains possible that two counteracting phenomena (one when detecting the error, the other when updating in long-term memory) may have been at play, and that the strong impact anisomycin infusion had on the general responding when the animals detected the prediction error may have obscured effects on long-term memory.

### 4.4. Anisomycin infusion in the BLA interferes with long-term memory of temporal updating

Experiment 4 assessed whether plasticity in the BLA is required for the long-term memory of updating temporal information, once a temporal prediction error has been normally detected. Rats underwent the same phases and procedures as in Experiment 3, except that both groups received the infusion (vehicle or anisomycin) immediately after, rather than before, the Error session.

**FIGURE 4:**
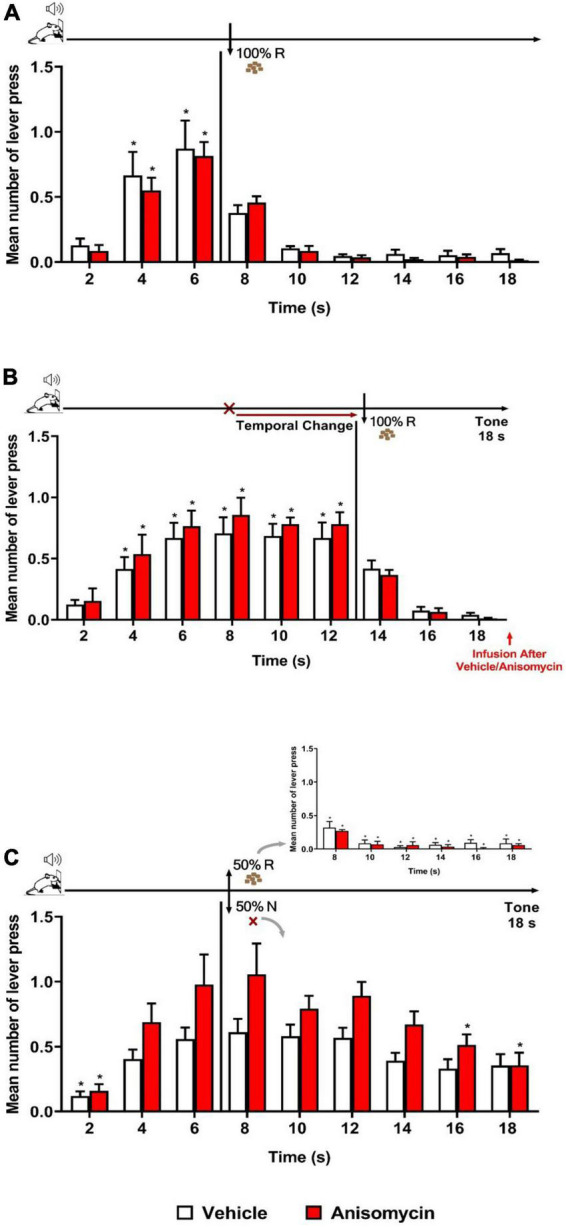
Anisomycin infusion after the Error session interfered with long-term memory of temporal updating. Responses average (+ SEM) during the 18 s tone, per 2 s, for Vehicle (White columns; *n* = 8) and Anisomycin (Red columns; *n* = 7) groups. **(A)** Retraining session during FI 6 s LH 12 s signaled schedule (*p < 0.05, intra-group differences from 2 s). **(B)** Error session of Temporal Change during FI 12 s LH 6 s signaled schedule during which the FI time was changed from 6 to 12 s to obtain the reward, and vehicle or anisomycin infusions were performed immediately after session (*p < 0.05, intra-group differences from 2 s). **(C)** Reinforcement omission effect (ROE) Test session under FI 6 s LH 12 s during which the reinforcement was omitted in 50% of trials (*p < 0.05, differences from 6 s). FI, fixed-interval; LH, limited hold; R, Reinforcement; N, non-reinforcement.

As expected, both groups (Vehicle and Anisomycin) presented similar performance during Training and Retraining sessions as in previous experiments. During Retraining (Figure 4A), there was a significant effect of time [*F* (8, 104) = 34.829, *p* < 0.001], with no significant effect of group [*F* (1, 13) = 0.113, *p* = 0.742], nor group × time interaction [F (8, 104) = 0.494, *p* = 0.858]. The analysis of the Error session (Figure 4B) showed that both Vehicle and Anisomycin groups updated similarly their responses to the new schedule of reinforcement (FI 12 s LH 6 s), as there were no significant effect of group [*F* (1, 13) = 0.375, *p* = 0.551], nor group × time interaction [*F* (8, 104) = 0.524, *p* = 0.836], but a significant effect of time [*F* (8, 104) = 36.583, *p* < 0.001].

The ROE test performed 24 h later showed that the anisomycin infusion after the Error session impaired the temporal behavior at long-term (Figure 4C). The ANOVA revealed a significant group × time interaction [*F* (8, 104) = 2.040, *p* < 0.05] and a significant effect of time [*F* (8, 104) = 17.309, *p* < 0.001], but no significant group effect [*F* (1, 13) = 4.247, *p* = 0.060]. The significant interaction points to differential temporal patterns of lever pressing between the two groups. *Post hoc* analyses showed that the number of lever presses increased significantly from 6 s when compared to time 2 s, for the Vehicle group, whereas it was increased from 4 s for the Anisomycin group. After 6 s (FI value), both groups expressed the ROE [with responses rates higher after non-reinforcement (N) than after reinforcement (R)]. During the *N* trials, the animals from the Vehicle group kept their lever presses at a high level for the remaining duration of the tone [no significant difference between the time 6 s and any of the following times (8, 10, 12, 14, 16, and 18 s)], whereas the animals of the Anisomycin group significantly reduced their lever pressing from 16 s onward (16 and 18 s), with a maximum expectancy around the FI (8 s) time. These results show that the anisomycin, when infused after the Error session, interfered with the long-term memory of the temporal updating experienced the day before during the Error session.

## 5. Discussion

The present study aimed at assessing whether basolateral amygdala nuclei (BLA) are involved in updating long-term memory in an appetitive operant conditioning after an unexpected delay in the time of reward’s availability (temporal prediction error). Our experimental design allowed us to study not only the expectancy during the stimulus period but also the post event behavior after reward or non-reward arrival has passed.

After Training, in which animals demonstrated temporal expectancy for the reward in a FI 6 s LH 12 s schedule of reinforcement (Black et al., 1972; Judice-Daher et al., 2011, 2012; Bueno et al., 2012; Tavares et al., 2014, 2019), a session with temporal prediction error generated a memory updating of reward expectancy to a new temporal schedule. The prediction error, either due to the temporal change or to the omission of reward, increased in the BLA the activation of a protein (Arc) that is critical for memory formation. Furthermore, anisomycin, an amnesic agent, infused in the BLA interfered with the updating and its storage in long-term memory.

### 5.1. BLA activation and prediction error

Many studies have shown the involvement of amygdala neurons, such as BLA, in prediction error detection processes. In particular, BLA neurons increase their firing rates when a surprising change is detected (Roesch et al., 2010, 2012; Tye et al., 2010; Schultz, 2015). For example, studies have demonstrated changes in neuronal activity in the amygdala at the omission of the US in non-human primates (Belova et al., 2007) and in rodents (Herry et al., 2007; Calu et al., 2010; Johansen et al., 2010a,b; Roesch et al., 2010). Belova et al. (2007) showed that prediction error detection modulated BLA firing rates, with some neurons increasing or decreasing their firing rate in response to the omission of the US whether appetitive or aversive.

In the present study, both the reinforcement omission and the temporal change of reward increased Arc activation, a gene regulator of protein synthesis-dependent forms of synaptic plasticity and memory storage (Guzowski et al., 2005; Bramham et al., 2010; Shepherd and Bear, 2011), in the BLA at the same level. Interestingly, the delay, from 6 to 12 s, in reward’s availability during the Error session results in an omission of reward at the moment when it was expected to arrive (i.e., at 6 s). It thus opens the possibility that the increased Arc activation observed in the Temporal change group was caused by that indirect omission of reward, rather than by the temporal change. However, a recent study using a Pavlovian aversive conditioning in rats has demonstrated Arc activation in basal amygdala after a change from long to short delay in the time of US arrival, thus producing no omission before the US arrival (Dallérac et al., 2017). Moreover, Bermudez et al. (2012) demonstrated that the reward signals of amygdala neurons represented the temporal expectations of reward, showing that the amygdala is involved in a temporal process for reward. Thus, it is possible that the Arc activations we observed here were related to both processes, linked in our case, temporal change and reward omission.

When the animals received anisomycin infusion in the BLA before the Error session (when the time of reward availability was changed from 6 to 12 s), they presented lower response rates than those in the Vehicle group. Similar results were found in rats with amygdala lesions (Tavares et al., 2014). Tavares et al. (2014) found that there was an overall decrease in the response rates only when the omission was introduced. Studies have demonstrated an important involvement of BLA in the formation and use of expectancies of reinforcers in devaluation procedures (Balleine et al., 2003; Blundell et al., 2003; Holland and Gallagher, 2004). Thus, it is possible that the anisomycin could have acted in processes related to the temporal omission competing with the temporal memory updating.

However, Dallérac et al. (2017) demonstrated that, when the CS-US time interval is changed, the basal amygdala network is involved in the maintenance of temporal expectancy to the initial/old time, and that extinction of this old expectancy is faster when the amygdala is inhibited. In fact, the general decrease in responding for the Anisomycin group might relate to the responses distribution, more concentrated toward 12 s, suggesting a better adaptation to the new learning. Thus, the anisomycin infusion into BLA could have disrupted the old memory from Training, facilitating the learning to the new temporal association. Such interpretation converges with a previous study reporting that infusion of anisomycin into the dorsal striatum, which belongs to a common functional network with the amygdala underlying temporal expectancy and interval timing, did not prevent rapid learning of a new time of reinforcement arrival in an appetitive instrumental peak interval paradigm (MacDonald et al., 2012).

### 5.2. Consolidation vs. reconsolidation

When the anisomycin was infused in the BLA after the Error session, leaving the error detection and behavioral adaptation intact, our results show that it disrupted the long-term (24 h) memory of the temporal updating. Although the differences in responding never reached significance between Vehicle and Anisomycin groups, intra-group analyses showed that, at Test (24 h after Error session), the animals responded differently in their temporal pattern depending on whether plasticity had been blocked, or not, in the BLA during the consolidation phase after the Error session. As expected, the animals from the Vehicle group demonstrated a memory of the temporal change experienced during the Error session the day before keeping their lever presses at a high level for the remaining duration of the tone, showing thus a temporal performance similar as the “Temporal Change” group from Experiment 1. In contrast, animals in the Anisomycin group presented a decrease in responding after the FI 6 s time, with a significant decay before the end of the duration of the tone, thus a temporal dynamic of behavior similar to the one of the “No Change” group in Experiment 1. The time course of temporal expectation expressed 24 h after the Error session in the Anisomycin group indicated a lack of memory of the temporal change the animals had detected during the Error session, and thus a disrupted stabilization in long-term memory of the temporal updating (consolidation process). These results indicate that plasticity in the BLA underlie long-term memory of a temporal updating.

Protein synthesis inhibitors can affect both consolidation and reconsolidation processes (Boccia et al., 2004; Wang et al., 2005; Duvarci et al., 2008). In our study, the anisomycin could have thus disrupted the memory for the initial FI time (reconsolidation), rather than the learning of the new FI time (consolidation). For example, Wang et al. (2005) showed that intra-amygdala infusions of anisomycin, whether given after the initial devaluation or after a second devaluation session, abolished the changes in the value of the food reward produced by incentive learning. This study provides direct evidence that instrumental incentive learning depends on protein synthesis within the BLA for both consolidation and reconsolidation and extends the demonstrations of protein synthesis-dependent reconsolidation to reward-related memories. However, research on consolidation and reconsolidation often uses models of single or few learning trials because they allow the analysis of the time course of changes that occur after initiating a learning trace (Fernández et al., 2016). Earlier studies have shown that reconsolidation occurs only under specific constrains, including memory characteristics (Milekic and Alberini, 2002; Suzuki et al., 2004) and experimental retrieval parameters (e.g., conditional context, duration, retrieval schedule (Auber et al., 2013; Reichelt and Lee, 2013; Dunbar and Taylor, 2017).

Reconsolidation of Pavlovian conditioned memories seems to require a minimum amount of non-reinforced CS exposure, and non-reinforced reactivation sessions often succeed in destabilizing Pavlovian memories (Lee J. L. et al., 2006; Milton et al., 2008; Reichelt and Lee, 2013; Exton-McGuinness et al., 2015). In instrumental tasks, the outcome is not clear. In some reports, non-reinforced reactivation sessions failed to lead destabilization and subsequent reconsolidation of instrumental memories (Hernandez et al., 2002; Hernandez and Kelley, 2004; Mierzejewski et al., 2009; Exton-McGuinness et al., 2014). However, other studies have shown that changes in the reinforcement contingency during a reactivation session impair reconsolidation of instrumental memory (Exton-McGuinness et al., 2014; Tedesco et al., 2014; Exton-McGuinness and Lee, 2015).

It is important to highlight that the time spent in the operating condition and/or the number of lever presses performed during retrieval is critical. A retrieval session that is too weak, for example, will not reactivate the memory trace, preventing its turn to instability. Conversely, excessively long phase durations or active lever presses may turn retrieval into a new phase that encodes a context-specific association competition (Bouton, 2004). Thus, as proposed by Exton-McGuinness et al. (2015), the variation of reward contingency during memory reactivation could be a successful trigger for memory destabilization, carrying enough unpredictability to induce a prediction error sufficient for instrumental memory destabilization. For example, in the Exton-McGuinness et al. (2014)’s study, after 10 days of Training in a fixed-ratio-1 (FR1) schedule of reinforcement, rats were submitted to a Reactivation session with a maximum of 20 rewards. They found that memory was destabilized and disrupted by systemic administration of MK-801 when administered prior to the switch to a variable (variable-ratio-20, VR20), but not under a fixed-ratio schedule of reinforcement. The disruption of reconsolidation resulted in a reduction of lever pressing performance at long-term and diminished the sensitivity of behavior to contingency change.

In the present study to assess the temporal change updating in one single operant session, we used 20 reinforced trials during reactivation (Error session), which could have induced a destabilization of memory and its subsequent reconsolidation, but could have also created a new trace involving learning processes. As proposed by Alberini (2011), two traces are present and both are in an unstable state when a new encoding appears after a memory reactivation: one mediates the reconsolidation of the reactivated trace in a non-asymptotic phase, and the other expresses a possible new encoded trace that goes through a new consolidation process. In our experiment, it is interesting to note that the anisomycin infusion after 20 reinforced trials in the new temporal association during the Error session returned the memory performance similar to the training level during the Test session, suggesting that the original trace had remained stable. Similar results have been reported in other studies that used multiple trial learning tasks to investigate the effects of amnesic treatments on memory consolidation (Meiri and Rosenblum, 1998; Luft et al., 2004; Touzani et al., 2007; Alberini, 2011).

Thus, although further experiments are needed to specifically address this issue, our results provide evidence that the anisomycin in the BLA had interfered only with the consolidation process of the new trace (temporal memory updating), and not provoked destabilization and consequent reconsolidation of the initially trained memory of operant conditioning, in neither its associative, nor temporal components.

## 6. Conclusion

In the present study, we demonstrated an involvement of BLA when the animal detects a change in temporal and reward contingencies, and its function in long-term memory of temporal updating. In summary, using a behavioral protocol that enables a specific assessment of temporal processes in an appetitive operant conditioning paradigm, the current investigation provides molecular and behavioral evidence of the BLA involvement in the temporal prediction error and in the long-lasting memory of time. The most important differences between studies of instrumental memory reconsolidation and consolidation appear to be the conditions under which memories are reactivated which controls whether consolidation and/or reconsolidation occurs (Alberini, 2011; Exton-McGuinness et al., 2015; Kida, 2020; Piva et al., 2020). The consolidation and reconsolidation of appetitive instrumental memory are topics of growing interest for the development of laboratory studies and pilot investigations in the clinic (Piva et al., 2020). Finding the parameters for reactivation is a key to successful memory destabilization, and temporal processing appears to be essential to explore these processes in the amygdala.

## Data availability statement

The raw data supporting the conclusions of this article will be made available by the authors, without undue reservation.

## Ethics statement

This animal study was reviewed and approved by the Ethics Committee N^°^59. All procedures were in accordance with the guidelines of the EU, CNRS, and the French Agricultural and Forestry Ministry (2010/63/UE, French decree article R-214-89).

## Author contributions

TT was responsible for the theoretical formulation of the research, for the study design, for collecting, analysis and interpretation of the data, and wrote the first and final version of the manuscript. VD was responsible for the theoretical formulation of the research, study design, interpretation of data, and collaborated in writing the final version of the manuscript. JB was responsible for the theoretical formulation of the research, collaborated in the interpretation of data, and in the writing of the final version of the manuscript. All authors contributed to the article and approved the submitted version.
